# Can Major Public Health Emergencies Affect Changes in International Oil Prices?

**DOI:** 10.3390/ijerph182412955

**Published:** 2021-12-08

**Authors:** An Cheng, Tonghui Chen, Guogang Jiang, Xinru Han

**Affiliations:** 1 Wu Jinglian School of Economics, Changzhou University, Changzhou 213159, China; chengan@cczu.edu.cn (A.C.); jiangguogang@cczu.edu.cn (G.J.); 2 Jiangsu Energy Strategy Research Base, Changzhou University, Changzhou 213159, China; 3 Institute of Agricultural Economics and Development, Chinese Academy of Agricultural Sciences, Beijing 100081, China; chentonghui@caas.cn

**Keywords:** public health emergency of international concern, international oil price, COVID-19, US dollar index, product partition model

## Abstract

In order to deepen the understanding of the impact of major public health emergencies on the oil market and to enhance the risk response capability, this study analyzed the logical relationship between major public health emergencies and international oil price changes, identified the change points, and calculated the probability of abrupt changes to international oil prices. Based on monthly data during six major public health emergencies from 2009 to 2020, this study built a product partition model. The results show that only the influenza A (H1N1) and COVID-19 pandemics were significant reasons for abrupt changes in international oil prices. Furthermore, the wild poliovirus epidemic, the Ebola epidemic, the Zika epidemic, and the Ebola epidemic in the Democratic Republic of the Congo had limited effects. Overall, the outbreak of a Public Health Emergency of International Concern (PHEIC) in major global economies has a more pronounced impact on international oil prices.

## 1. Introduction

As global industrialization continued to increase, economic development became increasingly dependent on oil [1,2]. International oil prices have also gradually become an important factor affecting the world economy, receiving widespread attention from academia and industry [3]. Thus, economists have expended efforts in explaining how oil prices are determined. In the long run, oil prices are determined by demand and supply. In the short run, previous research has examined other factors that may contribute to fluctuations of oil prices, such as military conflicts, war, geopolitics, terrorism, and macroeconomic-related or policy-related uncertainties [4,5,6,7,8,9]. Based on an analysis of all major oil price fluctuations between 1973 and 2014, Baumeister and Kilian evaluated the impact of exogenous factors such as the war in Iran and the global financial crisis of 2008 on the fluctuations in oil prices [1]. Chesney et al. found that sudden major natural disaster events could create significant shocks to financial markets [10]. Hartmann et al. studied the linkages between asset markets in the G5 (US, West Germany, UK, France, and Japan) that were in crisis and found the possibility of a simultaneous crash between the stock markets of each country [11]. White et al. adopted a VAR model to analyze the risk spillover effects in financial markets and found that shocks from crisis events could transmit risks in financial markets [12].

Since January 2020, an outbreak of coronavirus disease 2019 (COVID-19) has spread rapidly worldwide, leading to many business shutdowns, job losses, and store closures, as well as a reduction in social consumption levels, a weaker world economy, and a significant drop in international oil demand [13,14,15,16]. Due to the combination of the failed negotiations between OPEC and Russia to cut oil production and the COVID-19 outbreak, the price of the Western Texas Intermediate (WTI) crude oil futures contract fell rapidly, from approximately 60 USD/bbl in early 2020 to as low as 6.50 USD/bbl. The combination of the COVID-19 outbreak and dramatic changes in international oil prices has caused incalculable damage to the world economy [17]. 

Before the 20th century, humanity had long lacked effective means to cure infectious diseases [18]. In 1929, penicillin could treat infectious diseases that were caused by bacteria for the first time [19]. Subsequently, with the continuous advancement of medical and health care in society, the means of human resistance to infectious diseases have become increasingly abundant [20]. However, after the new millennium, global integration and the increasing exchange activities between the international community have significantly increased the spread of major international public health emergencies [21]. The World Health Organization (WHO) issued the International Health Regulations in 2007, which defined the concept of a Public Health Emergency of International Concern (PHEIC) [22]. Until 1 May 2020, six international public health emergencies had been declared by the WHO (Table 1): influenza A (H1N1) in 2009, the wild poliovirus epidemic in 2014, the Ebola epidemic in West Africa in 2014, the Zika epidemic in 2016, the Ebola epidemic in the Democratic Republic of the Congo (DRC) in 2019, and the COVID-19 pandemic in January 2020 [23].

Evidence has shown that infectious diseases threaten the survival and development of human society [24], and economists have also examined the impact of infectious diseases such as SARS on oil prices, as they may cause panic among investors and cut off the supply chain [25,26]. For example, Akhtaruzzaman et al. investigated the risk of oil price volatility in major global industries during the COVID-19 pandemic [27]. A positive oil price shock could benefit the oil supply industry but negatively affect the oil demand industry and the financial sector. Mensi et al. found that the oil market operated significantly less efficiently during the COVID-19 pandemic and the decline in oil prices, suggesting that this large-scale epidemic outbreak had a significant negative impact on the efficiency of the oil market operations [28]. 

Therefore, it is necessary to explore the relevance between the PHEIC and international oil prices. Such work can benefit government decision making, improve the risk warning system for major epidemics, and enhance the emergency management of enterprises and institutions in the future. However, most of the existing literature focuses on the effects of a single major public health emergency on international oil prices. A comprehensive review of the relevance between major international public health emergencies and international oil prices over time is lacking.

This paper advances the research frontier by investigating the logical relationship between major public health emergencies and international oil prices, estimating the abruptly changing characteristics of international oil prices during the six PHEIC events since 2009 using the product partition model (PPM) [29], and exploring the long- and short-term effects of epidemics on international oil prices. 

## 2. Deducing the Logical Relationship

Major public health emergencies of international concern are characterized by high infectiousness, difficulty in prevention and control, and high mortality. As soon as an epidemic occurs, it may adversely affect market confidence, oil supply and demand, etc., which, in turn, influences international oil prices [30,31,32,33,34].

Specifically, when a PHEIC breaks out, it will cast a shadow on the future economic development of the affected countries and regions, resulting in high market pressures, which have severely adverse effects on investor confidence. If the epidemic spreads to major economies, it may affect international oil demand and the regular operation of the oil industry, which will sharply increase investors’ expectations of uncertain risks in international oil prices [35,36,37,38,39]. Indeed, as it is affected by market sentiment, the US dollar index may fluctuate sharply. Since the US dollar index strongly correlates with oil prices, the fluctuation will further impact oil prices [40,41,42,43,44,45,46]. On the one hand, if the PHEIC is only of limited influence and short duration, and if there are effective measures to prevent and control it, its influence on oil price trends will gradually wane and disappear. On the other hand, when the PHEIC has a large impact range and a long duration, as well as if the prevention and control measures are inadequate, it might have a long-term impact on the international oil price trends by affecting supply and demand, as well as trade, storage, and transportation of oil [17,25,47,48,49].

## 3. Methods and Data

### 3.1. Methods

The product partition model (PPM) was used to calculate the monthly probability of abrupt changes in international oil prices. The US dollar index, global oil production, and oil consumption in OECD countries were selected as explanatory variables to analyze the relationship between major public health emergencies and abrupt changes in international oil prices and to derive the impacts of major public health emergencies on international oil price changes. The main reasons for applying the PPM are as follows: There are insufficient measures to separately estimate the impact of a major public health emergency on international oil prices. The PPM can effectively identify the probability of abrupt changes in monthly international oil prices, which makes it possible to infer the causes of sudden changes in oil prices from the results of the changes.

The PPM is a dynamic model that is used to analyze the change points in time series. It is assumed that the number of change points is unknown; then, the change points are identified, and the probability of changes and the posterior mean are tested. Barry and Hartigan were the first to apply the PPM to analyze change points using Bayesian analysis [29]. The PPM-based change point analysis technique has been introduced into the research of finance and prices [50,51,52,53].

It is assumed that $x_{1},x_{2}\cdots x_{n}$ is a set of known time series, and the index set is I ={1,2,⋯,n}. It is assumed that $\rho \;=\left\{i_{0},i_{1},\ldots ,i_{b}\right\}$ is randomly diverged from I ($0=i_{0}<i_{1}<\cdots <i_{b}=\;n)$; random variable *B* represents the number of regions in ρ). The original time series were divided into b subseries as $x_{\left[i_{r-1},i_{r}\right]}={\left(x_{i_{r-1}},\cdots ,x_{i_{r}}\right)}^{\prime }$, (r =1,⋯,b). $c_{\left[ij\right]}$ represents the prior tightness in [ij]={i+1,…,j} , and i*,*j∈I∪{0}, i < j. $c_{\left[ij\right]}$ represents the similarity between internal observations in $x_{\left[ij\right]}$. The prior tightness of [ij] is

(1) $$c_{\left[ij\right]}=\{\begin{array}{c} p{\left(1-p\right)}^{j-i-1}\;j<n\\ {\left(1-p\right)}^{j-i-1}\;j=n \end{array}$$

For all i,j∈I,i < j,$\;c_{\left[ij\right]}$ represents the possibility of an abrupt change in j after i. Equation (1) shows that the time series of change points are discrete and independent; that is, there is no correlation between continuous change points.

The marginal distribution densities of the time series are $f_{1}\left(x_{1}{|\theta }_{1}\right)$,⋯,$f_{n}\left(x_{n}{|\theta }_{n}\right)$, where $\theta_{1}$,$\theta_{2}$,⋯,$\theta_{n}$ are unknown parameters. In interval ρ, for $i_{r-1}<\;i\leq i_{r}$, the parameter is $\theta_{i}=\theta_{\left[i_{r-1},i_{r}\right]}$, where $\theta_{\left[i_{0}i_{1}\right]}$,⋯,$\theta_{\left[i_{b-1}i_{b}\right]}$ are all independent. Thus, the prior distribution of $\theta_{\left[ij\right]}$, namely, $\pi_{\left[ij\right]}\left(\theta \right)$,$\;\theta \in \Theta_{\left[ij\right]}$, $\Theta_{\left[ij\right]}$ represents parameter space. Therefore, time series random variables $(x_{1},x_{2},\cdots ,x_{n};\rho )$ obey the PPM.

### 3.2. Data

The monthly data regarding the Western Texas Intermediate (WTI) crude oil futures contracts listed on the New York Mercantile Exchange (NYMEX), the US dollar index (DXY, March 1973 = 100) obtained from the Intercontinental Exchange, and the global oil production and the oil consumption of OECD countries published by the Energy Information Administration (EIA) were used to analyze the change points. Monthly data were used for analysis because the effect of major international events on international oil prices usually lasts for weeks or even months. Thus, monthly data can better test the persistence of the impact on international oil prices. Daily or weekly data cannot reflect the medium- and long-term changes in international oil prices, while the sample size of quarterly or annual data is too small.

Following previous studies, we used the daily closing prices of continuous futures contracts of the NYMEX WTI crude oil [54,55]. As defined by Huang and Huang, continuous contracts refer to those contracts of the current transaction closest to the delivery month, and they will change as the delivery month approaches [54]. For the analysis, the interval range of the sample for international oil prices was from January 2009 to December 2020. The WHO announced a PHEIC in April 2009, meaning that obtaining samples from January 2009 ensured that the operating cycle of international oil prices coincided with the six PHEIC events.

In order to more effectively measure the probability of abrupt changes in international oil prices, the tolerance threshold for the probability of abrupt changes in international oil prices was set to 0.3, based on the relevant existing literature [56]. There is a higher probability of abrupt changes in international oil prices when the probability of sudden changes in international oil prices is greater than or equal to 0.3; when the probability of sudden changes in international oil prices is less than 0.3, there is a lower probability of abrupt changes in international oil prices.

## 4. Results

**Figure 1:**
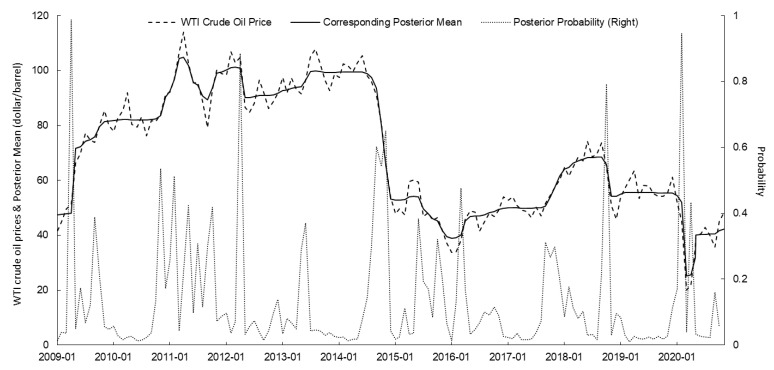
PPM calculation results of WTI crude oil prices and corresponding posterior mean and posterior probability since 2009 (source: NYMEX).

**Figure 2:**
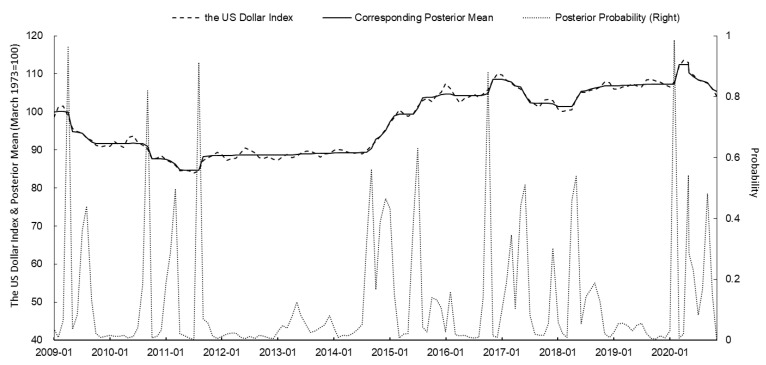
PPM calculation results of the US dollar index and corresponding posterior mean and posterior probability since 2009 (source: Intercontinental Exchange).

**Figure 3:**
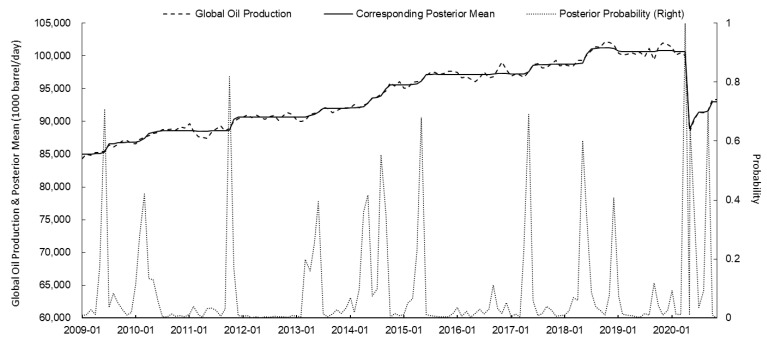
PPM calculation results of global oil production and corresponding posterior mean and posterior probability since 2009 (source: EIA).

**Figure 4:**
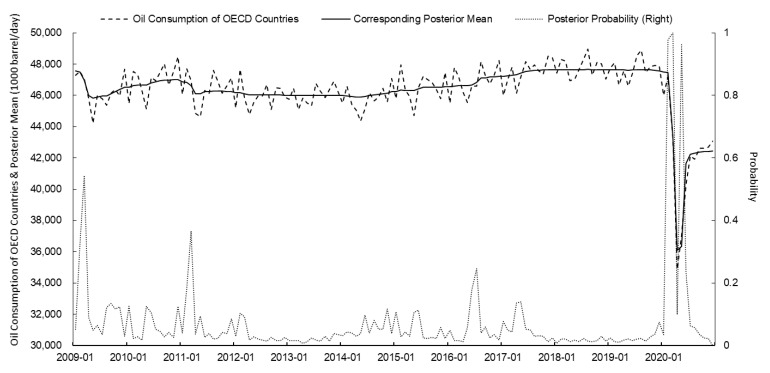
PPM calculation results of oil consumption of OECD countries and corresponding posterior mean and posterior probability since 2009 (source: EIA).

Based on the PPM, the change points of the WTI crude oil futures price, the US dollar index, the global oil production, and oil consumption of OECD countries from January 2009 to December 2020 were identified. The posterior mean and posterior probability were calculated using the *bcp* package of R. The results are shown in Figure 1, Figure 2, Figure 3 and Figure 4 and Table 2.

As shown in Table 2, among the six PHEIC events that have been announced by the WHO since 2009, the posterior probability of abrupt changes in WTI oil prices was higher than 0.3 in the following events: the influenza A (H1N1) pandemic in 2009; the Ebola epidemic in 2014; the Zika epidemic in 2016; the COVID-19 pandemic that began in January 2020. However, during the wild poliovirus epidemic in 2014 and the Ebola epidemic in DRC in July 2019, the posterior probability of abrupt changes in WTI oil prices was less than 0.3. Therefore, it is necessary to analyze the changes in international oil prices during the six PHEIC events separately.

### 4.1. The Influenza A (H1N1) Pandemic and Oil Price Changes

In April 2009, the influenza A (H1N1) pandemic first broke out in the US and Mexico and then spread to the rest of the world [57]. On 25 April, the WHO officially declared the influenza A (H1N1) pandemic as a PHEIC event. In general, the outbreak of the influenza A (H1N1) pandemic increased the probability of abrupt changes in WTI oil prices. Since the US implemented the quantitative easing policy at the end of 2008, the US dollar has continued to depreciate. The OPEC implemented the crude oil production limit in 2009. These two policies have caused a continuous increase in international crude oil prices [58,59]. When the influenza A (H1N1) pandemic spread across the world, the market panic was intensified. Investors rushed to seek safe-haven targets, and international crude oil futures were one of their natural choices. As shown in Figure 1 and Figure 2, and Table 2, the posterior probability of the abrupt change in the US dollar index in April 2009 (when the WHO officially declared the influenza A (H1N1) pandemic as a PHEIC event) reached 0.962, and the US dollar index fell sharply in May. The posterior probability of the abrupt change in WTI oil prices in April 2009 was as high as 0.988, and WTI oil prices rose sharply in May. Clearly, the global pandemic of influenza A (H1N1) exacerbated market concerns about future economic trends, promoted greater market risk aversion, and increased the price of WTI oil.

### 4.2. The Wild Poliovirus Epidemic and Oil Price Changes

The wild poliovirus is highly contagious and primarily affects children, but adults with low levels of antibody may also be infected [60]. As the adult prevalence rate in the Middle East and Central Africa increased sharply, the WHO officially declared the wild poliovirus epidemic a PHEIC event on 5 May 2014. Based on Table 2, while the wild poliovirus epidemic ravaged the Middle East and Central Africa, and the posterior probability of an abrupt change in global oil production that month was 0.416, the probability of a significant change in WTI oil prices that same month was 0.018. Therefore, the possibility of WTI oil prices changing in the future was low, i.e., the impact of the wild poliovirus epidemic on WTI oil prices was minimal.

### 4.3. The Ebola Epidemic and Oil Price Changes

In March 2014, the first human case of Ebola virus infection was discovered in Guinea [61]. In the following months, the Ebola epidemic broke out in various West African countries. On 8 August, the WHO declared the Ebola epidemic to be a PHEIC event. In that period, the US began to attract manufacturing back to the country in order to encourage the recovery of the economy and the strengthening of the dollar. Specifically, in August and September 2014, the posterior probability of an abrupt change in the US dollar index reached 0.316 and 0.562, respectively. The posterior mean of an abrupt change in the US dollar index rose sharply (Figure 2 and Table 2). Additionally, in August and September 2014, the posterior probability of an abrupt change in global oil supply reached 0.554 and 0.354, respectively. The posterior mean increased significantly, indicating a significant increase in global oil supply (Figure 3 and Table 2). The two factors above, acting in combination, caused a significant downward trend in WTI oil prices. In August and September 2014, the posterior probabilities of sudden changes in WTI oil prices were 0.304 and 0.602, respectively (Table 2). Although the Ebola epidemic was declared as a PHEIC event during that period, this virus mainly broke out in West African countries. Thus, its influence on oil supply and demand and market investment sentiment was relatively limited. Therefore, the Ebola virus is considered to have had a minimal effect on international oil prices. 

### 4.4. The Zika Epidemic and Oil Price Changes

Zika virus was first identified in Brazil in May 2015, and this less lethal but prevalent virus continued to spread in Brazil and other countries around the world [62]. On 18 February 2016, the WHO declared the Zika epidemic as a PHEIC event. Since the beginning of 2016, the supply and demand in the international oil market had been relatively stable, and the world had been in a relatively loose monetary environment. However, due to the severe cold weather, US crude oil and refined oil inventories fell sharply to their lowest point of the year, resulting in a significant short-term upward trend in international oil prices. In March 2016, the posterior probability of a sudden change in WTI oil price reached 0.476 (Table 2). The spread of Zika in Brazil and other countries did not have a significant impact on normal oil production, demand, or trade, nor did it cause significant changes in market sentiment. Therefore, it is believed that the Zika virus had a limited impact on international oil price fluctuation.

### 4.5. The Ebola Epidemic in DRC and Oil Price Changes

In 2018, the second Ebola epidemic began in the Democratic Republic of Congo (DRC) [63]. As the outbreak spread to other countries, the WHO declared the Ebola epidemic in DHR as a PHEIC on 17 July 2019. As shown in Table 2, during the Ebola epidemic in DRC, the posterior probabilities of abrupt changes in WTI oil price, US dollar index, global oil production, and the oil consumption of OECD countries were all below 0.3, indicating that the international oil price trend was relatively stable during this period. The possibility of various factors, including the Ebola virus, causing an abrupt change in WTI oil prices is low. Therefore, the impact of the Ebola epidemic in DRC on international oil prices was minimal.

### 4.6. The COVID-19 Pandemic and Oil Price Changes

On 31 January 2020, the WHO officially declared the COVID-19 outbreak as a PHEIC. COVID-19 spread rapidly throughout the world at the beginning of its outbreak as a result of its rapid and widespread transmission and the difficulty of its prevention and control [64]. According to statistics from Johns Hopkins University, as of 31 May 2020, there had been over 6 million confirmed COVID-19 diagnoses and more than 360,000 deaths worldwide, over 60% of which were from Europe and the US [65]. The impact of COVID-19 is enormous. Major countries worldwide suspended work and limited production in response to the effects of the epidemic, causing plummeting global crude oil demand and pessimistic market sentiment [66,67]. Meanwhile, the quantitative easing policies of major economies led to a sharp drop in international oil prices. As shown in Figure 1, Figure 2 and Figure 4, and Table 2, in February 2020, the posterior probability of a sudden change in the oil consumption of OECD countries reached 0.978, the posterior probability of a sudden change in the dollar index reached 0.986, and the posterior probability of a sudden change in WTI oil prices reached 0.946. Throughout the world, the COVID-19 pandemic affected the global economy, resulting in a sharp drop in international oil prices to some extent.

## 5. Discussion

Considering that the PHEIC announcement date can be at different times of the month, and the spread of the epidemic around the world does not always occur simultaneously, the epidemic could be delayed in some countries or regions. To assess the robustness of the research conclusions, the test period for measuring the abrupt changes in international oil prices was extended by one month based on Table 2. Specifically, the posterior probability of each factor at the PHEIC announcement date of the month and two months afterward is reported in Table A1. Results show that there is no significant change in the test results, compared with Table 2. This indicates that the results in Table 2 are robust.

**Figure 5:**
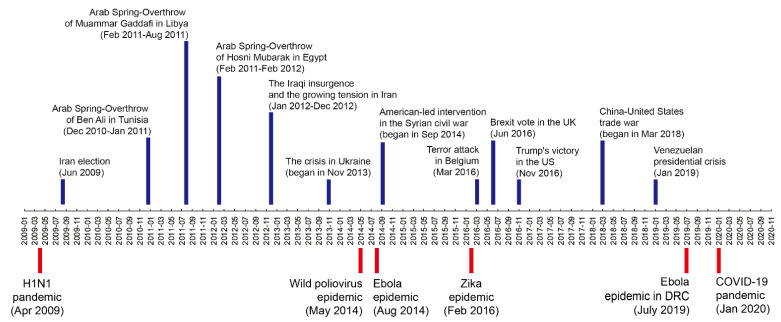
Important events in the Middle East and other major economies since 2009.

The previous study has shown that military conflicts, war, geopolitics, terrorism, and policy-related uncertainties may also contribute to fluctuations in oil prices [4,5,6,7,8,9]. Therefore, there may be other factors influencing the results of this study. In light of the fact that after the outbreak of the epidemic, major economies will formulate macroeconomic policies, and these policies will also have an effect on oil prices. To demonstrate the robustness of our findings, we also examined whether non-policy-related events, such as war, occurred during the research period by looking at the annual top 10 stories from BBC, NBC, AP, and other major media organizations. As shown in Figure 5, there are no obvious parallels between the time of the PHEIC announcements and the time of the important events in the Middle East and other major economies. Thus, our results in Table 2 are robust.

By combining quantitative and qualitative research, this article expands the current understanding of the impact of epidemic emergencies on international oil prices and can serve as a practical and policy-relevant guide. Firstly, the PHEIC event will have significant effects on international oil prices, resulting in an increase in systemic risks in the petroleum and petrochemical industries. As a result, the petroleum and petrochemical industry should attach great importance to the risk of oil price changes due to the impact of the epidemic and use oil price changes in international markets to hedge and protect themselves against risks in a timely manner. Secondly, to advance excellent research on PHEIC considering the wide variations in scope, duration, and severity of outbreaks, industry and academia should develop measurement and prediction models that will allow them to measure and predict the disease’s effect on historical data. An evaluation of the theoretical basis and a reliable foundation for improving risk early warning capabilities for the outbreak of PHEIC events should be discussed.

However, the research we conducted had a significant limitation that cannot be ignored. Considering that the process of estimating the impact of multiple PHEIC events on international oil price changes is derived from identifying abrupt changes in international oil prices during six PHEIC events, the results are subjective. Therefore, it will be necessary to investigate the use of quantitative tools to separate the degree of impact of previous PHEIC events from their effects on international crude oil prices in the future.

## 6. Conclusions and Insights

This study aimed to study the impact of multiple PHEIC on international oil price changes. The PPM was adopted to identify abrupt changes in international oil prices during six PHEIC events, based on monthly data from 2009 to 2020. Considering the changes in the US dollar index, oil production, and oil consumption, the reasons for the sudden change in international oil prices were analyzed. The results showed that four out of the six PHEIC events announced by the WHO since 2009 had posterior probabilities of abrupt changes in WTI oil prices that were over 0.3. Thus, only the global PHEIC events, the influenza A (H1N1) pandemic and the COVID-19 pandemic, had a more significant impact on market sentiment, and oil supply and demand, making them important contributors to the sudden changes in international oil prices during their outbreaks. The more localized PHEIC events, i.e., the wild poliovirus epidemic, the Ebola epidemic, the Zika epidemic, and the Ebola epidemic in DRC, had limited effects on sudden changes in international oil prices. In general, the outbreak of global PHEIC events in major economies in the world could cause more significant uncertainty in the international oil market and have a more noticeable impact on international oil prices.

According to this study, the PPM model can be used to identify post-mortem structural changes induced by major health crises. There are two main significances and values of this study. First, a forecasting model can be constructed by using several major variables that could change over time in order to predict the future trend of international oil prices. Second, it serves to provide a valuable reference for the formulation of future policies. The article suggests that the impact of the H1N1 and COVID-19 pandemics on market sentiment and international oil prices is similar. Hence, when the COVID-19 pandemic broke out, existing policies and measures against the impact of the H1N1 pandemic were emphatically put into practice. Furthermore, it is anticipated that a successful response to the COVID-19 and H1N1 pandemics will provide important policy recommendations for the future.

## Figures and Tables

**Table 1:** Major infectious disease outbreaks classified as PHEIC by the WHO since 2007.

Scheme	Outbreak Time	Epidemic Name
1	2009.3	Influenza A (H1N1) pandemic
2	2014.5	Wild poliovirus epidemic
3	2014.8	Ebola epidemic
4	2016.2	Zika epidemic
5	2019.7	Ebola epidemic in DRC
6	2020.1	COVID-19

Source: WHO.

**Table 2:** Changes in WTI oil price, US dollar index, global oil production, and oil consumption of OECD countries during PHEIC.

PHEIC	PHEIC Announcement Date	The Investigation Time of Abrupt Changes in Oil Prices	Posterior Probability of Abrupt Changes in Oil Prices	Posterior Probability of Abrupt Changes in the US Dollar Index	Posterior Probability of Abrupt Changes in Global Oil Production	Posterior Probability of Abrupt Changes in Oil Consumption of OECD Countries
The influenza A (H1N1) pandemic	2009.4.25	2009.4	0.988 *	0.962 *	0.012	0.090
		2009.5	0.050	0.036	0.174	0.048
The wild poliovirus epidemic	2014.5.5	2014.5	0.018	0.022	0.416 *	0.038
		2014.6	0.078	0.034	0.074	0.098
The Ebola epidemic	2014.8.8	2014.8	0.304 *	0.316 *	0.554 *	0.080
		2014.9	0.602 *	0.562 *	0.354 *	0.052
The Zika epidemic	2016.2.18	2016.2	0.128	0.160	0.006	0.016
		2016.3	0.476 *	0.018	0.022	0.016
The Ebola epidemic in DRC	2019.7.17	2019.7	0.024	0.056	0.016	0.020
		2019.8	0.018	0.022	0.010	0.022
COVID-19	2020.1.31	2020.1	0.172	0.030	0.092	0.034
		2020.2	0.946 *	0.986 *	0.014	0.978 *

Note: * indicates that the posterior probability of the abrupt change in international oil price is greater than 0.3; in view of the lag effect of the impact of a PHEIC on the international oil market, the posterior probability of the abrupt changes in each factor in the same and following month of a PHEIC announcement was selected for reporting.

## Data Availability

The data presented in this study are available upon request from the corresponding author.

## Appendix A

**Table A1:** Changes in WTI oil price, US dollar index, global oil production, and oil consumption of OECD countries during PHEIC.

PHEIC	PHEIC Announcement Date	The Investigation Time of Abrupt Changes in Oil Prices	Posterior Probability of Abrupt Changes in Oil Prices	Posterior Probability of Abrupt Changes in the US Dollar Index	Posterior Probability of Abrupt Changes in Global Oil Production	Posterior Probability of Abrupt Changes in Oil Consumption of OECD Countries
The influenza A (H1N1) pandemic	2009.4.25	2009.4	0.988 *	0.962 *	0.012	0.090
		2009.5	0.050	0.036	0.174	0.048
		2009.6	0.174	0.084	0.710 *	0.064
The wild poliovirus epidemic	2014.5.5	2014.5	0.018	0.022	0.416 *	0.038
		2014.6	0.078	0.034	0.074	0.098
		2014.7	0.144	0.052	0.098	0.040
The Ebola epidemic	2014.8.8	2014.8	0.304 *	0.316 *	0.554 *	0.080
		2014.9	0.602 *	0.562 *	0.354 *	0.052
		2014.10	0.542 *	0.166	0.002	0.052
The Zika epidemic	2016.2.18	2016.2	0.128	0.160	0.006	0.016
		2016.3	0.476 *	0.018	0.022	0.016
		2016.4	0.166	0.014	0.002	0.012
The Ebola epidemic in DRC	2019.7.17	2019.7	0.024	0.056	0.016	0.020
		2019.8	0.018	0.022	0.010	0.022
		2019.9	0.026	0.006	0.118	0.014
COVID-19	2020.1.31	2020.1	0.172	0.030	0.092	0.034
		2020.2	0.946 *	0.986 *	0.014	0.978 *
		2020.3	0.004	0.008	0.012	1.000 *

Note: * indicates that the posterior probability of the abrupt change in international oil price is greater than 0.3; in view of the lag effect of the impact of a PHEIC on the international oil market, the posterior probability of the abrupt changes in each factor in the same and following two months of a PHEIC announcement was selected for reporting.
